# Prediction of presurgical metabolic syndrome for gastric cancer‐specific mortality is more evident in smokers: The FIESTA study

**DOI:** 10.1002/cam4.5116

**Published:** 2022-08-26

**Authors:** Xinran Zhang, Dan Hu, Xiangling Deng, Jinxiu Lin, Xiongwei Zheng, Feng Peng, Fanqiang Meng, Wenquan Niu

**Affiliations:** ^1^ Institute of Clinical Medical Sciences, China‐Japan Friendship Hospital Beijing China; ^2^ Department of Pathology Fujian Cancer Hospital & Fujian Medical University Cancer Hospital Fuzhou China; ^3^ Department of Cardiology First Affiliated Hospital of Fujian Medical University Fuzhou China; ^4^ Department of General Surgery China‐Japan Friendship Hospital Beijing China

**Keywords:** gastric cancer, metabolic syndrome, prediction, risk score, smoking

## Abstract

**Backgrounds:**

We aimed to test whether the prediction of presurgical metabolic syndrome for postsurgical survival outcomes of gastric cancer hinges upon cigarette smoking status.

**Methods:**

This study is a part of the ongoing Fujian prospective investigation of cancer (FIESTA) study. Patients with gastric cancer received radical resection of primary gastric cancer between January 2000 and December 2010, with the latest follow‐up ended in December 2015. The 1:1 propensity score matching analysis was adopted to balance confounders between smokers and never‐smokers. Effect‐size estimates are expressed as hazard ratio (HR) with 95% confidence interval (CI). Model performance was evaluated using the Hosmer and Lemeshow test and 10‐fold cross‐validated area under the receiver operating characteristic curve (AUROC). Statistical analyses were completed with SAS software (v9.4).

**Results:**

Total 2779 patients with gastric cancer were analyzed, including 2223 smokers and 556 never‐smokers. Median follow‐up time was 45.6 months. Cigarette smoking was not associated with postsurgical survival differences. Presurgical metabolic syndrome complication was significantly associated with increased gastric cancer‐specific mortality in smokers (HR [95% CI]: 2.73 [1.53–4.89], *p* < 0.001), but not in never‐smokers. Relative excess risk due to interaction was estimated to be 2.43 (95% CI: 0.40–4.45). After constructing a risk assessment score, one unit increment was associated with 10% reduced risk of gastric cancer‐specific mortality (HR [95% CI]: 0.90 [0.88–0.91], *p* < 0.001), with 10‐fold cross‐validated AUROC being 0.82 (95% CI: 0.74–0.92).

**Conclusions:**

Our findings showed that the prediction of presurgical metabolic syndrome for gastric cancer‐specific mortality was more evident in smokers. Practically, this study provides evidence base for future personalized prediction and helped risk‐stratify gastric cancer patients who might experience serious postsurgical consequences.

## Graphical abstract

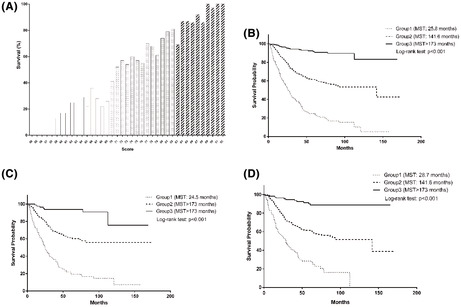

## 1 INTRODUCTION

Gastric cancer places a heavy burden on both individual and public health systems.^1^, ^2^ Globally, gastric cancer ranks as the sixth most common cancer and the third leading cause of cancer‐related mortality; about 1,089,103 new cases and 768,793 deaths were recorded in 2020.^3^ In China, newly diagnosed cases and deaths of gastric cancer were estimated to be 679,100 and 498,000 in 2015, respectively.^4^ Because clinical presentation of early gastric cancer is insidious, most patients are often diagnosed at an advanced stage and experience a poor prognosis as a consequence. Currently, surgery is the preferred treatment of choice for gastric cancer, but in some clinical settings, even after surgery, improvement in cancer survival is still poor.^5^ Hence, practical approaches that can accurately predict postsurgical survival outcomes of gastric cancer are critically needed.

Evidence is growing suggesting that cigarette smoking is a well‐known preventable factor of gastric cancer.^6^, ^7^, ^8^, ^9^ For example, in a systematic review and meta‐analysis of 42 articles, current smoking was significantly associated with 1.62‐ and 1.20‐fold increased risk of gastric cancer relative to never smoking in males and females, respectively. Epidemiologic studies have shown that presurgical smoking cessation for at least 2 weeks can lower the incidence of postsurgical complications in gastric cancer.^10^ Currently, little is known about whether postsurgical survival outcomes of gastric cancer differ between smokers and non‐smokers. To this point, many studies have revealed strong association of metabolic syndrome with occurrence and prognosis of cancer at many sites, including stomach.^11^, ^12^, ^13^, ^14^, ^15^ There is evidence that cigarette smoking and metabolic syndrome acted interactively in justifying early screening of colorectal cancer^16^ and increasing its recurrent risk in men.^17^ However, a literature search did not find any evidence in favor of the impact of cigarette smoking‐metabolic syndrome interaction on gastric cancer survival outcomes.

To fill this gap in knowledge, we aimed to test whether the prediction of presurgical metabolic syndrome for survival outcomes of gastric cancer hinged upon cigarette smoking status by analyzing the FIESTA (Fujian prospective investigation of cancer) database. Additionally, given the facts that the development and progression of gastric cancer involve multiple factors and contribution of individual factors is likely to be small, we attempted to construct a risk scoring system to further assess survival outcomes.

## 2 MATERIALS AND METHODS

### 2.1 Study design

The present study used data from the ongoing FIESTA study. The FIESTA study was initiated by Fujian Provincial Cancer Hospital in January 2000, with the aims to optimize presurgical risk factors and predict cancer‐specific mortality of common digestive system malignancies, including esophageal cancer, gastric cancer, and colorectal cancer.^11^, ^14^, ^15^, ^18^, ^19^, ^20^, ^21^ The Ethics Committee of Fujian Provincial Cancer Hospital approved the implementation of the FIESTA study (Approval Number: SQ2015‐070‐01).

### 2.2 Study patients

Total 3012 patients with gastric cancer who received radical resection of primary gastric cancer for the first time, underwent no presurgical and postsurgical chemotherapy/radiotherapy, and were consecutively recruited from January 2000 to December 2010, with the latest follow‐up ended on December 31, 2015, were eligible for inclusion, as previously described.^11^, ^13^, ^14^, ^15^, ^18^, ^19^, ^20^, ^21^, ^22^, ^23^, ^24^, ^25^, ^26^, ^27^, ^28^, ^29^ The reason for us to exclude patients with presurgical and postsurgical chemotherapy/radiotherapy is due to its significant impact on survival outcomes.

### 2.3 Tissue collection and diagnosis

Primary cancerous and adjacent normal tissue samples were collected during radical resection of primary gastric cancer, and they were fixed in 10% neutral‐buffered formalin and paraffin‐embedded using standard procedures. Presurgical biopsy and postsurgical pathological examination were used to confirm clinical diagnosis of gastric cancer.

### 2.4 Follow‐up assessment

Data were collected prospectively. Follow‐up interval was 6 months to 12 months after radical resection of primary gastric cancer. Patients who failed to appear or missed appointments at scheduled time were contacted them by phone calls or postal mails. All patients were followed up from initial admission after the surgery since January 2000 to death or last follow‐up visit before December 31, 2015, whichever occurred first.

### 2.5 Metabolic syndrome

In this study, metabolic syndrome was defined according to the criteria set forth by the Chinese Diabetes Society in 2004.^30^ In detail, a person is diagnosed to have metabolic syndrome when he/she has at least three of following criteria: (i) obesity: body mass index (BMI) ≥ 25 kg/m^2^; (ii) hyperglycemia: fasting blood glucose ≥6.1 mmol/L or 2‐h plasma glucose ≥7.8 mmol/L or previously diagnosed diabetes; (iii) hypertension: systolic/diastolic blood pressure ≥ 140/90 mm Hg or antihypertensive therapy; (iv) dyslipidemia: triglycerides ≥1.7 mmol/L or high‐density lipoprotein cholesterol (HDLC) <0.9 mmol/L in males or <1.0 mmol/L in females.

### 2.6 Demographic and clinicopathologic characteristics

All patients were invited to complete a self‐designed questionnaire to obtain demographic data at baseline, including age, gender, ABO blood type, smoking status, drinking status, and family cancer history. Recorded age referred to age at the time of initially receiving radical resection of primary gastric cancer. Smoking status was categorized as never, former, and current smoking, with the latter two categories combined as ever smoking. Similarly, drinking status was categorized as never, former, and current alcohol drinking, with the latter two categories combined as ever drinking. Family cancer history was recorded if one or more direct relatives are diagnosed with cancer (except non‐melanoma skin cancer) within three generations. Body height and weight were taken on patients in light clothing and without shoes, and they were used to calculate BMI. Blood pressure was measured in a seated position strictly according to the standard protocol recommended by the American Heart Association.^31^

Fasting (overnight at least 8 h) venous blood samples were collected at the morning of receiving radical resection of primary gastric cancer. Serum concentrations of glucose, plasma triglycerides, and total cholesterol, HDLC, and low‐density lipoprotein cholesterol (LDLC) were measured using standard procedures. An automated glucose oxidase method was used to assay fasting blood glucose.

In addition, routine blood indexes were recorded, including neutrophil, lymphocyte, monocyte, eosinophil, basophil, white blood cell count, red blood cell count, hemoglobin, red cell distribution width, and platelet count by the SYSMEX XE‐2100 Automatic Blood Cell Analyzer (Sysmex, Kobe, Japan). Interval from blood drawing to biochemical detection was less than 4 h according to standard procedures. Based on these indexes, neutrophil‐to‐lymphocyte ratio (NLR), platelet‐to‐lymphocyte ratio (PLR), lymphocyte‐to‐monocyte ratio (LMR), and monocyte‐to‐red blood cell count ratio (MRR) were derived accordingly.

Clinicopathologic characteristics including TNM stage (I, II, III, and IV according to the 7th Edition of the UICC/AJCC TNM Staging System^32^), tumor size, depth of invasion, regional lymph node metastasis, distant metastasis, Lauren's classification, and embolus were abstracted from medical charts and/or pathological reports.

### 2.7 Statistical analyses

Kaplan–Meier curves were used to display cumulative survival rates against follow‐up time, and Log‐rank tests were used to compare rate differences. The t‐test, Mann–Whitney U test or χ^2^ test was used to compare the distributions of baseline characteristics between two groups, where appropriate.

A propensity score was derived from Logistic regression analysis, with cigarette smoking status (smoking: 1; never‐smoking: 0) as the dependent variable. Variables that were selected based on clinical judgment and statistical significance in univariate comparisons were incorporated into propensity models, including age, gender, drinking, BMI, and family cancer history. An 1:1 propensity score matching analysis was then conducted between the smoking and non‐smoking groups based on derived propensity score. Those with closest scores were selected as pairs. After propensity score matching, hazard ratio (HR) with 95% confidence interval (CI) for gastric cancer‐specific mortality was estimated using adjusted and unadjusted Cox proportional hazard models. Proportional hazards assumption was tested by Schoenfeld residuals.

Variables that showed statistical significance in both smokers and never‐smokers were chosen to develop a risk scoring system for gastric cancer mortality prediction. The optimal cut‐offs for continuous variables were selected based on survival tree analysis using the STREE program (http://c2s2.yale.edu/software/stree/). The risk score for each prognostic factor was calculated as dividing 173‐month survival rate by 10, according to the method recommended by Sarah Douglas and colleagues.^33^ Individual scores were summed to generate a total score, and all patients were divided into three groups (<25th quantile, 25th–75th quantile, and ≥75th quantile).

Goodness‐of‐fit of Cox proportional hazard models for the total risk score in predicting risk of gastric‐cancer specific mortality was evaluated by the Hosmer and Lemeshow test. In addition, to reduce the variability because of the random stratification into 10 strata, the data are randomly divided into 10 equal parts, and the model is developed on 9/10 of the data (training set) and then tested on 1/10 of the remaining data (testing set). Then, a 10‐fold cross‐validated prediction model was performed to estimate area under the receiver operating characteristic curve (AUROC).

Statistical analyses were completed by the SAS software, version 9.4 (SAS Institute Inc.), unless otherwise indicated.

## 3 RESULTS

### 3.1 Baseline characteristics

At the end of follow‐up, there were 1331 (44.19%) deaths from gastric cancer and 1681 survivors. Median follow‐up time of all patients was 44.1 months (range: 1.1 to 183.3 months). Only 2779 patients had complete data on cigarette smoking status, including 2223 smokers and 556 never‐smokers. The baseline characteristics of study participants are shown in Table S1.

In view of striking differences in some baseline characteristics, such as gender composition and alcohol consumption, an 1:1 propensity score matching analysis was conducted between smokers and never‐smokers to balance confounders, and the baseline characteristics of study participants after the propensity score matching analysis are shown in Table 1.

**TABLE 1:** Baseline characteristics of patients with gastric cancer after the propensity score matching analysis^a^

Characteristics	Smokers (*n* = 397)	Never‐smokers (*n* = 397)	*p* ^b^
Age at surgery (years)	60 (53–67)	60 (52–69)	0.699
Males, *n* (%)	384 (96.73)	385 (96.98)	0.839
Drinking, *n* (%)	29 (7.30)	28 (7.05)	0.891
The ABO blood type			
O	153 (38.54)	180 (45.34)	0.264
A	133 (33.50)	115 (28.97)	
B	85 (21.41)	80 (20.15)	
AB	26 (6.55)	22 (5.54)	
Family cancer history	58 (14.61)	57 (14.36)	0.920
Body mass index (kg/m^2^)	22.51 (20.42–24.57)	22.06 (20.50–23.88)	0.199
Tumor‐node‐metastasis stage			
I/II	109 (27.53)	123 (30.98)	0.285
III/IV	287 (72.47)	274 (69.02)	
Invasion depth			
T1/T2	61 (15.37)	92 (23.17)	0.005
T3/T4	336 (84.63)	305 (76.83)	
Regional lymph node metastasis			
N0	114 (28.72)	135 (34.01)	0.129
N1	125 (31.49)	126 (31.74)	
N2	124 (31.23)	116 (29.22)	
N3	34 (8.56)	20 (5.04)	
Distant metastasis			
Negative	356 (89.67)	354 (89.17)	0.818
Positive	41 (10.33)	43 (10.83)	
The Lauren's classification			
Intestinal type	177 (45.04)	164 (41.41)	0.304
Diffuse type	216 (54.96)	232 (58.59)	
Tumor embolus			
Negative	239 (60.81)	263 (66.41)	0.102
Positive	154 (39.19)	133 (33.59)	
Tumor size (cm)	5.0 (3.5–7.0)	4.5 (3.0–6.0)	<0.001
Number of regional lymph node metastasis	3.0 (0.0–8.0)	2.0 (0.0–7.0)	0.293

*Note*: Data are expressed as median (interquartile range) or count (percentage), where appropriate.

^a^ Propensity score matching model included age, gender, drinking, body mass index, and family cancer history.

^b^ *p* was calculated by the Mann–Whitney U test for continuous variables and the Chi‐square test for categorical variables.

### 3.2 Propensity score matching and overall risk prediction

After the propensity score matching analysis, Kaplan–Meier curves and Log‐rank tests failed to reveal any significance in cumulative survival rates for gastric cancer between smokers and never‐smokers. Schoenfeld residuals did not show major departures from the proportional hazards assumption.

After adjusting for these nonsignificant variables, NLR, PLR, HDLC, and hyperglycemia were found to be significantly associated with gastric cancer‐specific mortality in both smokers and never‐smokers (Table 2). Risk estimation for hyperglycemia was reinforced in smokers relative to never‐smokers (HR: 4.84 vs. 2.11, 95% CI: 3.53–6.64 vs. 1.51–2.96, *p*_*interaction*_ < 0.001). Smokers with hypertension had 1.65‐fold increased risk for gastric cancer‐specific mortality (HR: 1.65, 95% CI: 1.05–2.58), while never‐smokers with hypertension had 1.63‐fold increased risk (HR: 1.63, 95% CI: 1.09–2.45). Smokers with obesity (HR: 1.95, 95% CI: 1.13–3.37), dyslipidemia (HR: 1.77, 95% CI: 1.10 to 2.84) or metabolic syndrome (HR: 2.73, 95% CI: 1.53–4.79) showed a higher risk relative to smokers without, and interaction with never‐smokers was not significant for these variables (all *p*_*interaction*_ > 0.05) (Table 2).

**TABLE 2:** Risk estimates for cancer‐specific mortality by cigarette smoking status in patients with gastric cancer after the propensity score matching analysis

	Smokers				Never‐smokers				
Characteristics	Unadjusted HR (95% CI)	*p*	Adjusted HR (95% CI)	*p* *	Unadjusted HR (95% CI)	*p*	Adjusted HR (95% CI)	*p* *	*p*_*interaction*_ **
**Baseline characteristics**									
The ABO blood type									
O	Ref.		Ref.		Ref.		Ref.		
A	0.98 (0.68–1.40)	0.905	1.00 (0.69–1.44)	0.987	1.26 (0.89–1.8)	0.187	1.29 (0.90–1.84)	0.163	0.331
B	1.19 (0.80–1.77)	0.396	1.18 (0.78–1.76)	0.435	0.9 (0.58–1.38)	0.634	0.97 (0.62–1.50)	0.882	0.523
AB	1.28 (0.70–2.32)	0.426	1.30 (0.71–2.37)	0.396	1.13 (0.57–2.27)	0.712	1.19 (0.59–2.39)	0.623	0.851
Clinicopathologic characteristics									
Tumor‐node‐metastasis stage									
I/II	Ref.		Ref.		Ref.		Ref.		
III/I	5.47 (3.21–0.31)	<0.001	5.95 (3.48–10.19)	<0.001	5.21 (3.15–8.61)	<0.001	5.07 (3.06–8.39)	<0.001	0.670
Invasion depth									
T1/T2	Ref.		Ref.		Ref.		Ref.		
T3/T4	7.58 (3.11–18.45)	<0.001	8.06 (3.30–19.70)	<0.001	4.41 (2.50–7.77)	<0.001	4.36 (2.47–7.70)	<0.001	0.255
Regional lymph node metastasis									
N0	Ref.		Ref.		Ref.		Ref.		
N1	2.12 (1.28–3.53)	0.004	2.21 (1.33–3.69)	0.002	2.84 (1.75–4.60)	<0.001	2.86 (1.76–4.65)	<0.001	0.473
N2	4.66 (2.89–7.49)	<0.001	5.15 (3.18–8.32)	<0.001	5.02 (3.15–8.01)	<0.001	4.79 (2.99–7.67)	<0.001	0.833
N3	6.90 (3.89–12.24)	<0.001	7.92 (4.43–14.16)	<0.001	7.61 (4.07–14.2)	<0.001	8.57 (4.54–16.15)	<0.001	0.857
Distant metastasis									
Negative	Ref.		Ref.		Ref.		Ref.		
Positive	3.83 (2.66–5.51)	<0.001	4.19 (2.87–6.12)	<0.001	4.61 (3.21–6.63)	<0.001	4.70 (3.26–6.77)	<0.001	0.669
The Lauren's classification									
Intestinal type	Ref.		Ref.		Ref.		Ref.		
Diffuse type	1.89 (1.37–2.60)	<0.001	1.97 (1.43–2.73)	<0.001	2.02 (1.44–2.84)	<0.001	2.20 (1.57–3.10)	<0.001	0.645
Tumor embolus									
Negative	Ref.		Ref.		Ref.		Ref.		
Positive	2.18 (1.61–2.96)	<0.001	2.22 (1.63–3.01)	<0.001	1.75 (1.28–2.38)	<0.001	1.87 (1.36–2.56)	<0.001	0.445
Tumor size (cm)	1.21 (1.15–1.27)	<0.001	1.21 (1.16–1.28)	<0.001	1.19 (1.13–1.25)	<0.001	1.20 (1.14–1.27)	<0.001	0.824
Number of regional lymph node metastasis	1.06 (1.05–1.08)	<0.001	1.07 (1.05–1.09)	<0.001	1.08 (1.06–1.10)	<0.001	1.08 (1.06–1.10)	<0.001	0.488
**Laboratory biomarkers**									
Neutrophil (10^9^/L)	1.16 (1.07–1.25)	<0.001	1.16 (1.07–1.25)	<0.001	1.06 (1.00–1.13)	0.048	1.08 (1.01–1.15)	0.022	0.167
Lymphocyte (10^9^/L)	0.71 (0.57–0.90)	0.005	0.71 (0.57–0.90)	0.005	0.63 (0.47–0.85)	0.003	0.69 (0.52–0.92)	0.011	0.878
Monocyte (10^9^/L)	0.69 (0.37–1.26)	0.221	0.65 (0.35–1.21)	0.175	1.01 (0.36–2.81)	0.970	1.00 (0.38–2.66)	0.998	0.464
Eosinophil (10^9^/L)	0.52 (0.21–1.27)	0.151	0.55 (0.23–1.35)	0.193	0.74 (0.33–1.67)	0.480	0.69 (0.28–1.69)	0.413	0.725
Basophil (10^9^/L)	0.94 (0.05–17.78)	0.969	1.03 (0.05–20.85)	0.983	1.06 (0.01–106)	0.978	3.04 (0.03–329.79)	0.643	0.702
White blood cell count (10^9^/L)	1.08 (1.01–1.16)	0.033	1.08 (1.00–1.16)	0.039	1.02 (0.95–1.09)	0.460	1.04 (0.97–1.11)	0.250	0.461
Red blood cell count (10^9^/L)	0.96 (0.87–1.07)	0.446	0.96 (0.88–1.06)	0.459	0.96 (0.87–1.06)	0.425	0.97 (0.89–1.06)	0.547	0.874
Hemoglobin (g/L) (per 30+)	0.95 (0.91–1.00)	0.049	0.95 (0.91–1.00)	0.059	0.91 (0.87–0.96)	<0.001	0.93 (0.88–0.98)	0.004	0.560
Platelet count (10^9^/L) (per 100+)	1.19 (1.03–1.38)	0.021	1.21 (1.04–1.40)	0.015	0.96 (0.81–1.12)	0.585	1.00 (0.85–1.19)	0.963	0.096
Red cell distribution width (%)	1.00 (0.99–1.01)	0.861	1.00 (0.99–1.01)	0.880	1.00 (0.98–1.02)	0.838	1.00 (0.97–1.02)	0.691	1.000
NLR	1.01 (0.98–1.03)	0.488	1.01 (0.98–1.04)	0.442	1.10 (1.05–1.16)	<0.001	1.12 (1.06–1.18)	<0.001	0.001
PLR (per 150+)	1.03 (0.94–1.12)	0.573	1.03 (0.94–1.13)	0.477	1.46 (1.11–1.91)	0.006	1.50 (1.14–1.97)	0.004	0.008
LMR	0.91 (0.82–1.00)	0.055	0.91 (0.83–1.01)	0.068	0.93 (0.85–1.03)	0.930	0.96 (0.88–1.04)	0.302	0.416
MRR	0.85 (0.46–1.58)	0.605	0.84 (0.42–1.66)	0.612	0.93 (0.22–3.84)	0.914	0.97 (0.21–4.57)	0.973	0.867
**Metabolic indexes**									
Obesity	1.60 (1.14–2.24)	0.006	1.95 (1.13–3.37)	0.017	1.04 (0.70–1.55)	0.847	1.52 (0.83–2.76)	0.173	0.548
Hypertension	1.66 (1.06–2.57)	0.025	1.65 (1.05–2.58)	0.029	1.70 (1.16–2.48)	0.006	1.63 (1.09–2.45)	0.019	0.969
SBP (mm Hg) (per 10+)	1.14 (1.06–1.23)	0.001	1.13 (1.04–1.23)	0.003	1.10 (1.01–1.19)	0.024	1.08 (0.99–1.18)	0.100	0.465
DBP (mm Hg) (per 10+)	1.17 (1.02–1.33)	0.025	1.16 (1.01–1.33)	0.036	1.06 (0.93–1.22)	0.366	1.05 (0.91–1.21)	0.505	0.324
Dyslipidemia	1.72 (1.09–2.72)	0.020	1.77 (1.10–2.84)	0.018	2.83 (0.90–8.88)	0.075	3.49 (0.99–12.34)	0.052	0.323
HTC (mmol/L)	0.93 (0.80–1.08)	0.342	0.92 (0.79–1.07)	0.270	0.95 (0.79–1.13)	0.525	0.91 (0.76–1.09)	0.299	0.928
HTG (mmol/L)	1.23 (1.07–1.42)	0.004	1.21 (1.05–1.41)	0.011	1.49 (1.01–2.21)	0.046	1.47 (0.98–2.20)	0.065	0.375
LHDLC (mmol/L)	0.97 (0.83–1.15)	0.755	0.96 (0.81–1.13)	0.620	0.94 (0.77–1.14)	0.498	0.91 (0.75–1.10)	0.326	0.680
HLDLC (mmol/L)	0.43 (0.26–0.70)	0.001	0.43 (0.26–0.71)	<0.001	1.06 (0.63–1.77)	0.832	1.01 (0.60–1.71)	0.969	0.021
Hyperglycemia	4.80 (3.52–6.55)	<0.001	4.84 (3.53–6.64)	<0.001	2.25 (1.63–3.12)	<0.001	2.11 (1.51–2.96)	<0.001	<0.001
Fasting glucose (mmol/L)	1.34 (1.28–1.41)	<0.001	1.34 (1.28–1.41)	<0.001	1.14 (1.07–1.21)	<0.001	1.14 (1.07–1.21)	<0.001	<0.001
Metabolic syndrome	2.81 (1.65–4.78)	<0.001	2.73 (1.53–4.89)	<0.001	2.07 (1.01–4.21)	0.046	1.51 (0.66–3.45)	0.327	0.251

*Note*: Effect‐size estimates were calculated under the COX proportional hazards regression model. Abbreviations: 95% CI, 95% confidence interval; DBP, diastolic blood pressure; HLDLC, high low‐density lipoprotein‐cholesterol; HR, hazard ratio; HTC, high total cholesterol; HTG, hypertriglyceridemia; LHDLC, low high‐density lipoprotein cholesterol; LMR, lymphocyte‐to‐monocyte ratio; MRR, monocyte‐to‐red blood cell count ratio; NLR, neutrophil‐to‐lymphocyte ratio; PLR, platelet‐to‐lymphocyte ratio; SBP, systolic blood pressure.

* *p* was adjusted for age, gender, drinking, body mass index, and family cancer history

** *p*_*interaction*_ was calculated by the Z‐test.

HRs for gastric cancer‐specific mortality among never‐smokers with metabolic syndrome, smokers without metabolic syndrome, and smokers with metabolic syndrome were 1.59 (95% CI: 0.78–3.23), 1.07 (95% CI: 0.85–1.34), and 4.08 (2.57–6.49), respectively, compared with never‐smokers without metabolic syndrome (Table 3).

**TABLE 3:** Interaction analysis between metabolic syndrome and cigarette smoking on gastric cancer‐specific mortality

Metabolic syndrome	Smoking status	Cases	Total	HR (95% CI)	*p* *
Absence	Never smoking	156 (40.52)	385	Ref.	
Presence	Never smoking	9 (64.29)	14	1.59 (0.78–3.23)	0.204
Absence	Ever smoking	146 (40.11)	364	1.07 (0.85–1.34)	0.564
Presence	Ever smoking	25 (80.65)	31	4.08 (2.57–6.49)	<0.001
RERI	2.43 (0.40–4.45)				
AP	0.59 (0.28–0.91)				
SI	4.70 (0.78–28.48)				

*Note*: Effect‐size estimates were calculated under the COX proportional hazards regression model. Abbreviations: 95% CI, 95% confidence interval; AP, attributable proportion due to interaction; HR, hazard ratio; RERI, relative excess risk due to interaction; SI, synergy index.

* *p* was adjusted for age, gender, drinking, body mass index, and family cancer history.

Relative excess risk due to interaction (RERI) was estimated to be 2.43 (95% CI: 0.40–4.45), indicating that there was 2.43 relatively excess risk due to additive interaction (Table 3). About 59% of mortality due to smoking and metabolic syndrome was attributable to additive interaction (attributable proportion: 0.59, 95% CI: 0.28–0.91). Mortality risk in smokers with metabolic syndrome was 4.70 times as high as the sum of risk in participants exposed to each risk factor alone (synergy index: 4.70, 95% CI: 0.78–28.48) (Table 3).

### 3.3 Risk scoring system for gastric cancer‐specific mortality

**FIGURE 1:**
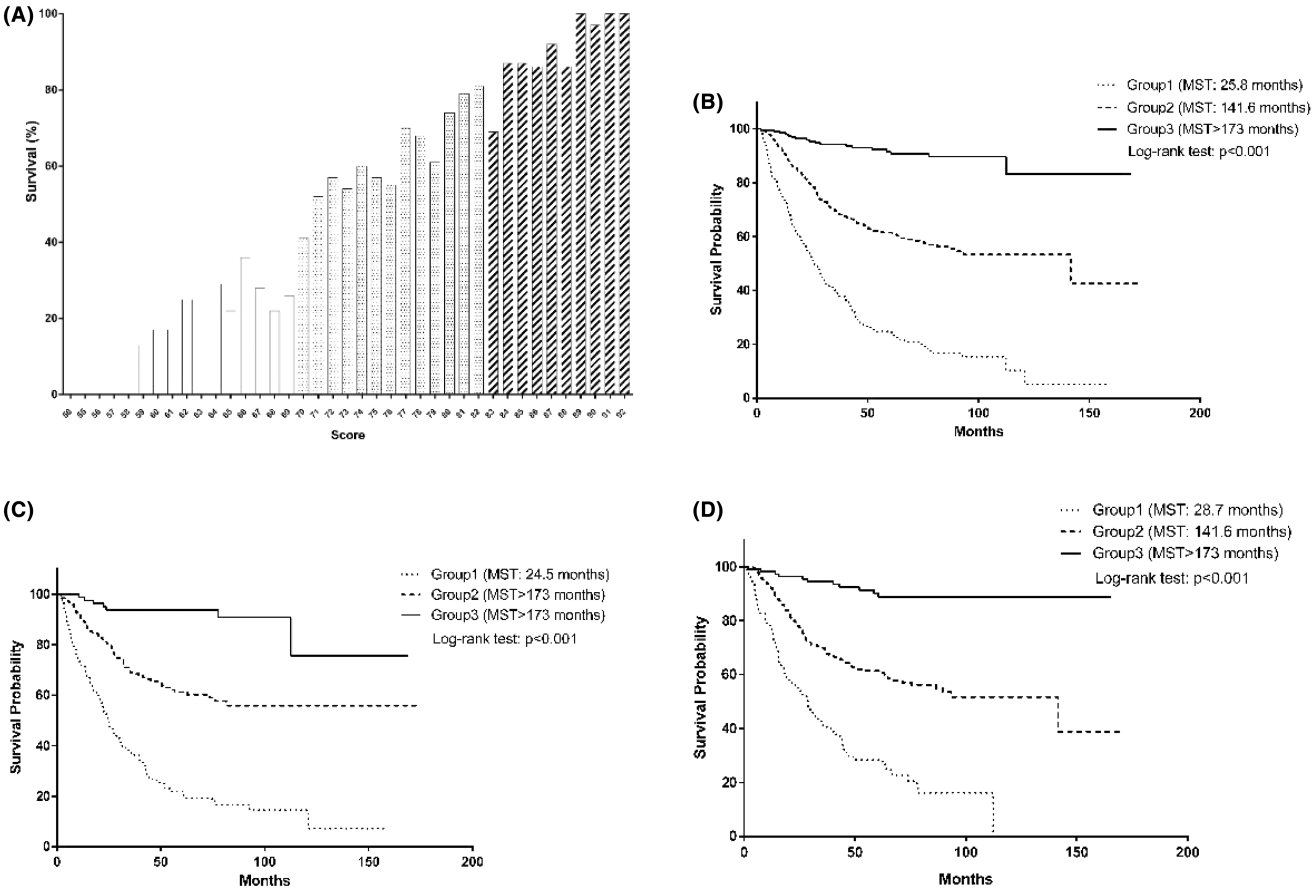
Distribution of the total risk score with postsurgical survival rate after follow‐up (A), and Kaplan–Meier curves by score groups in patients overall (B) and by cigarette smoking status (panel C in smokers and panel D in never‐smokers). MST, median survival time. Group 1 refers to patients with a total score ranging from 50 to 69; group 2 refers to patients with a total score ranging from 70 to 82; group 3 refers to patients with a total score ranging from 83 to 92.

Based on significant variables identified above, a risk scoring system was created to estimate survival rates of patients with gastric cancer. The distribution of this risk score is presented in Figure 1A. Survival tree analysis was used to select optimal cut‐off value of each continuous variable (Figure S1). The score for each prognostic variable was calculated as dividing 173‐month survival rate by 10 (Table 4).

**TABLE 4:** Overall survival rates at 173rd month after radical resection of primary gastric cancer and the corresponding smoking‐dependent risk scores

Characteristics	Number	Survival at 173rd month, %	Score values
Tumor‐node‐metastasis stage			
I/II	232	86	9
III/IV	561	46	5
Invasion depth			
T1/T2	153	88	9
T3/T4	641	50	5
Regional lymph node metastasis			
N0	249	82	8
N1	251	61	6
N2	240	38	4
N3	54	20	2
Distant metastasis			
Negative	710	64	6
Positive	84	8	1
The Lauren's classification			
Intestinal type	341	70	7
Diffuse type	448	49	5
Tumor embolus			
Negative	502	66	7
Positive	287	44	4
Tumor size (cm)			
Q1: ≤5.8	502	66	7
Q2: >5.8	292	43	4
Number of regional lymph node metastasis			
Q1: (~3.3)	439	74	7
Q2: (3.3–13)	278	42	4
Q3: (13~)	77	21	2
White blood cell count (10^9^/L)			
Q1: (~8.8)	697	59	6
Q2: (8.8~)	97	46	5
Hemoglobin (g/L)			
Q1: (~131)	417	49	5
Q2: (131–136)	87	59	6
Q3: (136~)	290	70	7
NLR			
Q1: (~1.7)	303	68	7
Q2: (1.7–2.3)	213	58	6
Q3: (2.3~)	278	46	5
PLR			
Q1: ≤199.4	656	60	6
Q2: >199.4	138	46	5
Metabolic syndrome			
No	747	60	6
Yes	45	24	2

Abbreviations: NLR, neutrophil‐to‐lymphocyte ratio; PLR, platelet‐to‐lymphocyte ratio.

Total score that represented sum of individual scores ranged from 50 to 92, and per‐score increment was associated with 10% reduced risk of gastric cancer‐specific mortality (HR: 0.90, 95% CI: 0.88–0.91) (Table 5). In addition, significance existed in both smokers (HR: 0.89, 95% CI: 0.87–0.91) and never‐smokers (HR: 0.90, 95% CI: 0.88–0.92).

**TABLE 5:** Risk estimates of the risk score for gastric cancer‐specific mortality

Groups	Adjusted HR (95% CI) in all patients	*p* *	Adjusted HR (95% CI) in smokers	*p* *	Adjusted HR (95% CI) in never‐smokers	*p* *
Overall	0.90 (0.88–0.91)	<0.001	0.89 (0.87–0.91)	<0.001	0.90 (0.88–0.92)	<0.001
Accuracy assessment						
Hosmer and Lemeshow test (*p*)	0.492		0.434		0.501	
10‐fold CV AUROC (95% CI), *p*	0.82 (0.74–0.92), <0.001		0.83 (0.75–0.93), <0.001		0.82 (0.74–0.93), <0.001	
Quantiles						
<25th quantile	Ref.		Ref.		Ref.	
25th–75th quantile	0.33 (0.27–0.42)	<0.001	0.31 (0.23–0.43)	<0.001	0.34 (0.24–0.47)	<0.001
>75th quantile	0.06 (0.04–0.10)	<0.001	0.06 (0.03–0.12)	<0.001	0.07 (0.04–0.13)	<0.001

*Note*: Effect‐size estimates were calculated under the COX proportional hazards regression models. Abbreviations: 95% CI, 95% confidence interval; AUROC, area under the receiver operating characteristic curve; CV, cross‐validation; HR, hazard ratio.

* *p* was adjusted for age, gender, drinking, body mass index, and family cancer history.

The goodness‐of‐fit of this total score for gastric cancer‐specific mortality was good as the probability of Hosmer and Lemeshow test was nonsignificant (>0.4) in patients overall and by cigarette smoking status (Table 5). Moreover, using a 10‐fold cross‐validation procedure, the AUROC was 0.82 (95% CI: 0.74–0.92), 0.83 (95% CI: 0.75–0.93), and 0.82 (0.74–0.93) in all patients, smokers, and never‐smokers, respectively (*p* < 0.001 for all) (Table 5).

Then, total score was classified into three groups based on their distributions among all study patients (score range: 50–69 for group 1; 70–82 for group 2; 83–92 for group 3). Kaplan–Meier curves of the three groups for cumulative survival against follow‐up time in all study patients, smokers, and non‐smokers are displayed in Figure 1B–D, respectively.

Survival rates were 22.90%, 60.26%, and 90.77% for group 1, group 2, and group 3, respectively. Relative to group 1, patients in group 2 (HR: 0.33, 95% CI: 0.27–0.42) and group 3 (HR: 0.06, 95% CI: 0.04–0.10) separately had 67% and 94% reduced risk of gastric cancer‐specific mortality after adjusting for confounders (Table 5). In addition, significance was consistently identified in both smokers and never‐smokers.

## 4 DISCUSSION

Our findings indicate that the prediction of presurgical metabolic syndrome for gastric cancer‐specific mortality is more evident in smokers. To facilitate clinical application, we constructed a comprehensive risk scoring system based on significant contributors of either smokers or never‐smokers, and interestingly found that this scoring system exhibited a strong discriminatory capability to predict postsurgical survival outcomes.

In the oncology field, several presurgical scoring systems, such as modified systemic inflammation score (modified SIS), prognostic nutritional index (PNI), and controlling nutritional status (CONUT) score,^34^, ^35^, ^36^, ^37^, ^38^ have been developed and gained notable success in predicting cancer mortality risk. For example, modified SIS that incorporated serum albumin and LMR was proposed as a useful prognostic tool for postsurgical survival in patients with gastric cancer.^34^ Both PNI score and CONUT score took nutritional status into consideration.^35^, ^36^, ^37^, ^38^ The CONUT score was useful for long‐term survival outcomes in gastric cancer patients at stage I‐II in Japanese.^39^ By contrast, the prognostic value of CONUT score was only found in Chinese gastric cancer patients at stage II‐III who underwent curative resection and adjuvant chemotherapy.^40^

More recently, we and other researchers have found that metabolic syndrome was associated with an increased risk of gastric cancer‐specific mortality.^11^, ^41^ Although our findings failed to support a contributory role of cigarette smoking in the prognosis of gastric cancer, we interestingly observed a divergent risk profile between smokers and never‐smokers, and a wide panel of significant risk factors in either smokers or never‐smokers were selected to develop a risk scoring system. Actually, this score had a better predictive ability of gastric cancer‐specific mortality. In order to create a powerful scoring system of prognostic measures for gastric cancer postsurgically, it is necessary to externally replicate this score in other ethnic groups.

The key finding of this study is the additive interaction between cigarette smoking and metabolic syndrome when predicting postsurgical gastric cancer‐specific mortality. Cigarette smoking is a well‐known and established risk factor for gastric cancer,^6^, ^42^, ^43^ and growing evidence indicates significant association between metabolic syndrome complication and worse gastric cancer survival.^11^ Currently, the relevance between cigarette smoking and metabolic syndrome in cancer prognosis remains controversial. For example, there was an additive impact of current smoking habits and metabolic syndrome complication on colorectal cancer recurrence risk,^13^ but there was no observable interaction between cigarette smoking and metabolic factors with bladder cancer.^44^ By far, no study has yet investigated potential interaction between cigarette smoking and metabolic syndrome on postsurgical gastric cancer survival. We, in this large cohort, observed a significant additive interaction between metabolic syndrome and cigarette smoking in predicting postsurgical gastric cancer‐specific mortality. In particular, the presence of metabolic syndrome and its adverse metabolic components were significantly associated with an increased risk of gastric cancer‐specific mortality in smokers, but no significance existed in never‐smokers except for hypertension and hyperglycemia. The mechanisms underlying the interaction between cigarette smoking and metabolic syndrome are not fully understood. It is plausible that this interaction is due to inflammation response. There is wide recognition that inflammation plays a crucial role in the development, progression, and recurrence of cancer.^45^ Metabolic syndrome contributes to cancer development and progression via multi‐ways, including inflammatory cytokines.^46^ Cigarette smoking can induce inflammation by oxidative stress or releasing TNF‐alpha.^47^, ^48^ Nevertheless, it is of clinical importance to prevent the development of presurgical metabolic syndrome as far as possible or treat it reasonable in time to avoid unexpected postsurgical consequences in patients with gastric cancer, especially among smokers.

Besides the clear strengths of this study, including the prospective design, large sample size, and long follow‐up, several limitations should be acknowledged. First, we recruited patients from a single center, which may limit the generalizability of our findings, although it facilitates consistency of evaluation and intervention. Second, we had data on cigarette smoking only, and other important smoking‐related indexes, such as pack‐years smoked and smoking cessation were not available. Additionally, information on perisurgical factors such as surgical procedure, lymphadenectomy and reconstruction, as well as subsequent management of metabolic syndrome after radical resection of primary gastric cancer was not available for us, which might introduce a prediction bias. Third, although we adopted the propensity score matching analysis to balance baseline confounders between smokers and never‐smokers, there are still other confounders without taking into consideration, such as *Helicobacter pylori*, an established risk factor for gastric cancer. Additionally, after the propensity score matching analysis, only 397 smokers and 397 never‐smokers were assessable in this study, which led to loss of statistical power. Fourth, only patients who received radical resection of primary gastric cancer, but no presurgical and postsurgical chemotherapy/radiotherapy were enrolled, which might limit the extrapolation of our findings to general patient groups. Additionally, our findings were exclusively derived from a southern Chinese population, and additional replications in other ethnic or racial groups are critical. Fifth, more male patients than female patients were enrolled in this cohort, which might restrict extrapolation to the more general populations.

## 5 CONCLUSIONS

Our findings indicate that the prediction of presurgical metabolic syndrome for gastric cancer‐specific mortality is more evident in smokers. Importantly, we have constructed a risk scoring system, which exhibited a strong discriminatory capability to predict postsurgical survival outcomes in patients with gastric cancer. Although this scoring system needs external validation, it has provided evidence base for future personalized prediction and helped risk‐stratify gastric cancer patients who might experience serious postsurgical consequences.

## AUTHOR CONTRIBUTION

DH, WN planned and designed the study, and directed its implementation; FP, DH, XZ drafted the protocol; DH, XZ obtained statutory and ethics approvals; DH, XZ contributed to data acquisition; XD, XZ, FP, WN conducted statistical analyses; XZ, XD, WN, DH, FP, XZ had access to all raw data; FP, DH, FM, WN did the data preparation and quality control; XZ, FM, WN wrote the manuscript. All authors read and approved the final manuscript prior to submission.

## FUNDING INFORMATION

There are no commercial interests or sources of financial or material support to declare.

## CONFLICT OF INTEREST

The authors declare that they have no competing interests, and all authors should confirm its accuracy.

## ETHICS APPROVAL AND CONSENT TO PARTICIPATE

All participants provided their written informed consent, and this study was approved by the Ethics Committee of Fujian Provincial Cancer Hospital.

## Supporting information


Appendix S1

## Data Availability

The datasets used during the current study are available from the corresponding authors upon reasonable request.
