# Supplementary material for: Prediction of presurgical metabolic syndrome for gastric cancer‐specific mortality is more evident in smokers: The FIESTA study
Source: Cancer Med. 2022 Aug 26;12(3):3419–32. doi: 10.1002/cam4.5116 (PMC9939207; doi:10.1002/cam4.5116)
Supplement: Supplementary file 1 — Appendix S1 [file CAM4-12-3419-s001.docx]

**SUPPLEMENTARY TABLE 1.** Baseline characteristics of cohort patients by cigarette smoking status before the propensity score matching analysis

| **Characteristics** | **Never-smokers**  **(n=556)** | **Smokers**  **(n=2,223)** | | ***p*^*^** |
| --- | --- | --- | --- | --- |
| Age at surgery (years) | 60 (53-67) | | 58 (50-67) | 0.003 |
| Males, n (%) | 538 (97.29) | | 1,522 (68.84) | <0.001 |
| Drinking, n (%) | 134 (24.23) | | 32 (1.45) | <0.001 |
| The ABO blood type |  | |  |  |
| O | 213 (38.52) | | 897 (40.57) | 0.395 |
| A | 178 (32.19) | | 640 (28.95) |  |
| B | 119 (21.52) | | 515 (23.29) |  |
| AB | 43 (7.78) | | 159 (7.19) |  |
| Family cancer history | 83 (15.01) | | 175 (7.91) | <0.001 |
| Body mass index (kg/m^2^) | 22.55 (20.45-24.61) | | 22.66 (20.75-24.91) | 0.097 |
| Tumor-node-metastasis stage |  | |  |  |
| I/II | 149 (26.85) | | 603 (27.17) | 0.877 |
| III/IV | 406 (73.15) | | 1,616 (72.83) |  |
| Invasion depth |  | |  |  |
| T1/T2 | 91 (16.37) | | 406 (18.27) | 0.295 |
| T3/T4 | 465 (83.63) | | 1816 (81.73) |  |
| Regional lymph node metastasis |  | |  |  |
| N0 | 151 (27.16) | | 592 (26.63) | 0.537 |
| N1 | 187 (33.63) | | 699 (31.44) |  |
| N2 | 170 (30.58) | | 751 (33.78) |  |
| N3 | 48 (8.63) | | 181 (8.14) |  |
| Distant metastasis |  | |  |  |
| Negative | 503 (90.47) | | 1,933 (87.03) | 0.027 |
| Positive | 53 (9.53) | | 288 (12.97) |  |
| The Lauren's classification |  | |  |  |
| Intestinal type | 237 (42.93) | | 834 (37.87) | 0.029 |
| Diffuse type | 315 (57.07) | | 1,368 (62.13) |  |
| Tumor embolus |  | |  |  |
| Negative | 332 (60.14) | | 1,348 (61.22) | 0.644 |
| Positive | 220 (39.86) | | 854 (38.78) |  |
| Tumor size (cm) | 5.0 (3.5-7.0) | | 5.0 (3.5-7.0) | 0.941 |
| Number of regional lymph node metastasis | 4.0 (0.0-8.0) | | 3.0 (0.0-8.0) | 0.160 |

Data are expressed as median (interquartile range) or count (percentage), where appropriate. **p* was calculated by the Mann-Whitney U test for continuous variables and the χ^2^ test for categorical variables.


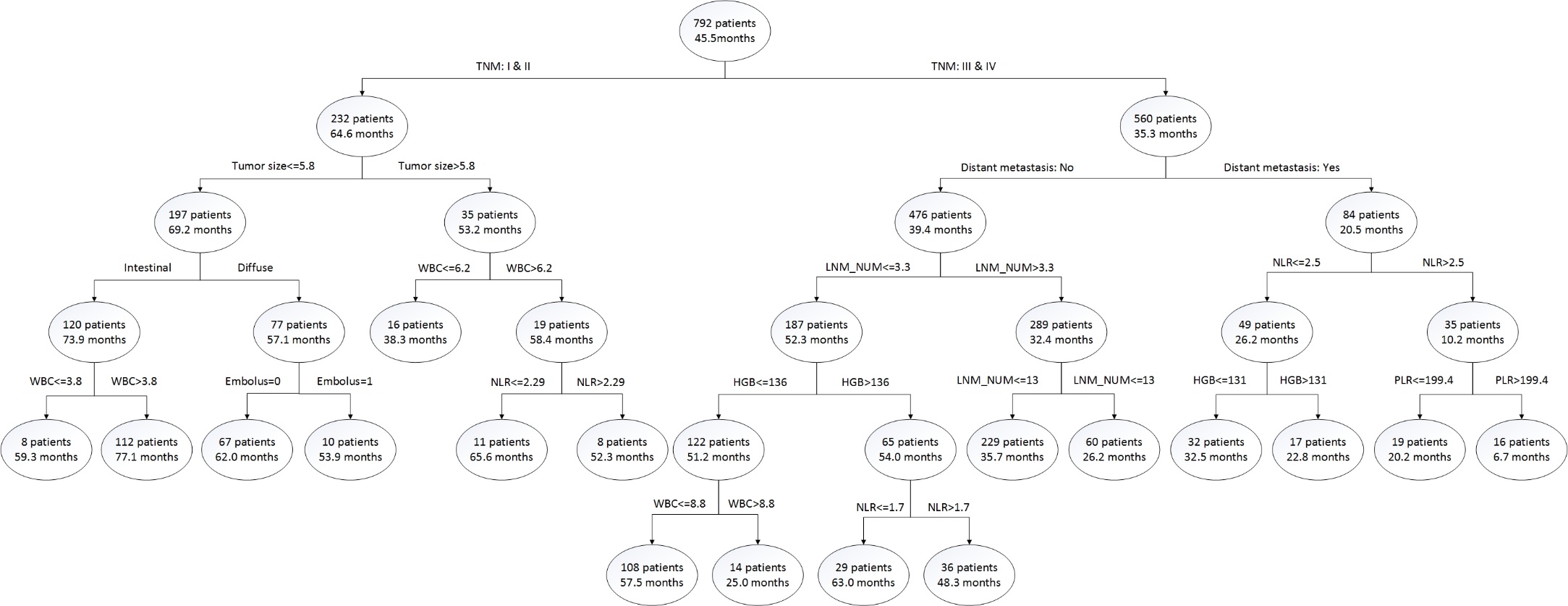


**SUPPLEMENTARY FIGURE 1.** Survival tree structure of blood routine parameters or derivates, and clinical characteristics on prognosis of gastric cancer-specific mortality. Abbreviations: TNM, tumor-node-metastasis stage; WBC: white blood cell count; NLR: neutrophil-to-lymphocyte ratio; LNM_NUM: number of regional lymph node metastasis; HGB, hemoglobin; PLR, platelet-to-lymphocyte ratio.
